# Health-Related Quality of Life among People Applying for Housing Adaptations: Associated Factors

**DOI:** 10.3390/ijerph15102130

**Published:** 2018-09-27

**Authors:** Lovisa Boström, Carlos Chiatti, Björg Thordardottir, Lisa Ekstam, Agneta Malmgren Fänge

**Affiliations:** 1 Department of Health Sciences, Faculty of Medicine, Lund University, P.O. Box 157, 221 00 Lund, Sweden; mev15lbo@student.lu.se (L.B.); carlos.chiatti@med.lu.se (C.C.); lisa.ekstam@med.lu.se (L.E.); 2 Department of Occupational Therapy, Prosthetics and Orthotics, Faculty of Health Sciences, OsloMet—Oslo Metropolitan University, P.O. Box 4 St. Olavs Plass, 0130 Oslo, Norway; bjorgt@oslomet.no

**Keywords:** ADL, disability, health, home, usability

## Abstract

Housing adaptations (HA) clients are a heterogeneous group of people with disabilities experiencing restricted performance and participation in everyday life. While health-related quality of life is a common and relevant outcome in health care research, associated factors among HA clients are largely unknown. Thus, the aim of this study was to investigate which factors are associated with health-related quality of life among HA clients in Sweden. The study has a cross-sectional design, using baseline data collected among 224 participants in three municipalities in Sweden. The main outcome was health-related quality of life measured by the EQ-5D. Factors investigated as potentially associated were age, sex, living conditions, cognitive impairment, usability of the home, activities of daily living (ADL) dependence, participation, and fear of falling. The associations were explored using multiple linear regression analysis. Younger age and higher dependence in ADL were associated with lower scores on the EQ-5D. The social aspect of usability in the home had a positive association with the EQ VAS. Results suggest that certain groups of HA clients might be at risk for low health-related quality of life. Knowledge of their characteristics can potentially improve development and implementation of tailored interventions aiming at increasing their health-related quality of life.

## 1. Introduction

Health-related quality of life is widely accepted as important outcomes for evaluating the effect of interventions, both on individual and societal level [1,2]. Apart from having proved to be a strong predictor of mortality [3,4], self-rated health is a valuable alternative indicator of a health condition [2]. Studies have shown that a variety of factors affect how people rate their own health. In general, personal factors such as higher age, being a woman, low income, low education level, living alone, and body functions and structure such as cognitive impairment and having a chronic disease are all related with worse scores on self-rated health assessments [5,6,7]. Mobility, disability, and high risk of falling are also known factors associated with lower self-rated health [8,9]. Falls are common among older people, and most falls occur in people’s own homes [10,11,12], as consequence of environmental factor hazards such as tripping over thresholds or loose rugs [11,12].

The ageing-in-place principle, aiming to allow people to remain living in their own homes while ageing, has been inspiring policy reform in Sweden and many other countries [13]. Several older adults aspire to remain in their homes for several personal and practical reasons. This desire is also beneficial from a societal point of view, as it relates to lower costs for the society, compared for example to the cost of nursing home placements [14]. In this respect, a factor contributing to successful ageing-in-place strategies is the design of the home environment. Environmental barriers and accessibility problems in the home or in the close neighborhood can indeed have a negative impact on activity and participation both inside and outside the home [15], which in turn may lead to reduced self-rated health and quality of life [16].

In Sweden, people experiencing activity limitations and dependence on other people due to the presence of physical environmental barriers in the home can apply to the municipality for a housing adaptation (HA) grant. The individual can submit a certification of his/her needs and an application for a HA grant, which the municipality can entirely or partly approve or reject. Unlike to what is the case in most countries, HA in Sweden are publicly funded, and regulated by the Housing Adaptations Act [17]. Approximately 73,000 HAs are funded in Sweden each year, to a total cost of 1.039 million Swedish kronor. Common adaptations granted were mounting of grab bars, installation of ramps, and adaptations in hygiene areas [18].

People applying for HA are a heterogeneous group, in terms of e.g., age, independence in activities of daily living, participation in social activities and living conditions [19,20]. Most of them are 70 years or older [18] with declining physical and cognitive capacity due to normal ageing, or diseases such as stroke and dementia. However, younger people with injuries or diseases belong to this population [20,21]. Though different in terms of sociodemographic, clinical, and functional characteristics, they share the common need for having their physical home environment adapted to live independently. While it is known that a home environment unfit for their needs can negatively impact their health [22], other factors related to the person, the activity and the home environment specifically associated with health-related quality of life among this population remains a largely uninvestigated area. Knowledge about such determinants might support the HA process by providing valuable information on those concomitant factors which could be addressed by the professionals working in the municipalities during their interventions to benefit this group of clients. This may influence the overall effectiveness of the HA intervention. To address the current research gaps, the aim of this study was to investigate which factors are associated with health-related quality of life among people applying for HAs in Sweden.

## 2. Materials and Methods

This study has a cross-sectional design. It draws on baseline data from a larger trial (ClinicalTrials.gov.NCT01960582), implemented among HA applicants in Southern Sweden [19], approved by the Ethical Review Board at Lund University (Dnr 2012/556).

### 2.1. Sampling and Participants

Three municipalities were included in the study. The selection is based on their number of inhabitants, their geographical dispersion, and their willingness to participate in the project. Individuals living in these municipalities applying for a HA were asked to participate in the study, if they met the following inclusion criteria: aged 20 years or older, living in the community and applying for a HA after contact with the municipality occupational therapist. Excluded were those living in sheltered housing and those not able to communicate in Swedish [19]. In total, 224 participants were included in this study.

### 2.2. Data Collection

Data were collected by occupational therapists during home visits before the start of the HA. The home visit took approximately 90 min and the data collection included both self-assessments and observations by means of standardized instruments and study specific questions. In two municipalities, the occupational therapists employed by the municipality gathered the data, and in one, the occupational therapist was employed by the project. They were especially trained in the methodology applied to the study to uniform the data collection process [19]. Data collection at baseline took place between March 2013 and December 2015.

### 2.3. Study Outcome Variables

The outcome variables of the study were measured using the EQ-5D, a standardized instrument developed by the EuroQol Group as a measure of health-related quality of life. The EQ-5D consists of a descriptive system assessing five dimensions of health, namely mobility, self-care, usual activities, pain/discomfort and anxiety/depression, and a vertical visual analogue scale, EQ VAS. In the version of the descriptive system used in this study, the EQ-5D-5L, the five dimensions are assessed on a five-graded ordinal scale [23]. The respondent’s scoring obtained on these dimensions can be converted to a single summary index number reflecting their preference compared to other health profiles, with a higher index score indicating better health profile. Respondents are then asked to rate their overall health on the day of the interview on the vertical visual analogue scale, EQ VAS, from 0 to 100, where 0 indicates the worst and 100 the best imaginable health. The EQ-5D has been tested extensively for validity and reliability [24,25]. In this study, we used separately both the index score obtained from the descriptive system and the ratings from the visual analogue scale, as two distinct but relevant measures of health-related quality of life among HA clients.

### 2.4. Other Study Variables

- Demographic data included age, sex and living conditions classified as “Living alone and having no close relationship with a partner”, “Living alone and having a close relationship with a partner”, “Living together with partner/family” and “Other”.
- Cognitive impairment was assessed using the Montreal Cognitive Assessment (MoCA), a screening tool including eight domains: short-term memory, visuospatial abilities, executive functions, attention, concentration, working memory, language and orientation in time and place. The maximum score is 30 points, with a lower score indicating a higher level of cognitive impairment. MoCA has been tested for validity and reliability [26].
- Usability of the home environment was assessed by a revised version of Usability In My Home (UIMH) instrument [27]. The revised version consists of 11 self- reported questions on how satisfied the individual is with the home environment in different activities, such as cleaning, cooking, leisure activities etc. There are five response alternatives for each question, reaching from “very unsatisfied” (1 point) to “very satisfied” (5 points). Those who did not perform the activity described in the item were coded with a 0. Higher sum score indicates higher satisfaction with the home usability. UIMH has been tested for validity and reliability [27], and a further validation is currently ongoing [28]. Three aspects of usability have been identified; “Self-care aspects” (compromising 5 items: going to the toilet, personal hygiene, preparing meals, preparing snacks, and moving around the home with/without a mobility device, score 5–25 points), “Social aspects” (compromising 3 items: socializing with family and friends in the home, contacting others via telephone/Skype, and watching TV/listening to radio, score 3–15 points) and “Leisure/outdoor aspects” (compromising 3 items: entering the house, picking up the mail, and engaging in hobbies and leisure activities in the home, 3–15 points) [20].
- ADL dependence was assessed using the ADL-staircase [29] compromising nine activities; feeding, mobility, using the toilet, dressing, bathing, cooking, transportation, shopping, and cleaning, and shows how independent the individual is in these situations. The response alternatives are “independent without difficulty”, “independent with difficulty”, “partly dependent” or “dependent”. The responses were summarized in a total score, 0–27, with a higher score indicating more problems. The ADL-staircase has been tested for validity and reliability [29].
- Participation frequency and satisfaction were assessed by means of eight study specific statements related to: (a) how frequently and (b) how satisfied they were with the following: having contact with others in their home, helping others, doing something outside their home with others, and doing something outside their home alone.
- Fear of falling was assessed with a dichotomous yes/no question.

### 2.5. Analysis Design and Statistics

Data on participant age showed a non-normal distribution and was therefore split into three age categories: category 1 (age < 75 years), category 2 (age 75–90 years) and category 3 (age > 90 years). Category 2 (75–90 years) was used as the reference category in the regression analysis.

Living conditions were reclassified into two response alternatives. “Living alone and having no close relationship with a partner”, “Living alone and having a close relationship with a partner” and “Other” were classified as “Living alone”. “Living together with partner/family” was classified as “Living together”. The scores from MoCA, ADL-staircase and UIMH (all three aspects) were reclassified from continuous to categorical variables. This was done to describe and visualize differences between groups. MoCA scores were classified into moderate, mild, or normal, and ADL-staircase and UIMH scores were divided into quartiles.

Descriptive statistics included independent T-tests and ANOVA examining the variables associations to health-related quality of life as measured separately by the EQ-5D-5L and the EQ VAS. Variables potentially associated with the outcomes (*p*-value below 0.2 at bivariate level) were then included in the multiple regression analysis by stepwise-forward selection, starting with variables that significantly differed between groups in the bivariate analysis. The independent variables were gender, age, living conditions, cognitive impairment, ADL dependence and usability of the home. We also tested the influence of items related to frequency and satisfaction with participation, and fear of falling but since they showed no potential association with the outcomes of interest, these variables were not included in the final analysis.

Associations with health-related quality of life were analyzed with multiple linear regression analysis using robust standard errors [30]. We used the EQ-5D-5L and the EQ VAS separately as the dependent variables of the regressions. Level of significance was set to *p* < 0.05. Post-estimation diagnostics was conducted by checking normality of residuals, the link test and measuring the information criteria of the models. Statistical analysis was performed using SPSS Statistics 25.0 (IBM Corporation, Armonk, NY, USA) and Stata 15.0 (Statacorp, College Station, TX, USA, 2015).

## 3. Results

The average self-rated health for the whole sample using the EQ VAS was 56.23 ± 20.89, while the mean EQ-5D-5L index score was 0.54 ± 0.25. The relationship between health-related quality of life and exposure variables at bivariate level are shown in Table 1. In the bivariate analysis, significant differences in self-rated health measured using the VAS score were found in relation to age, ADL dependence, and all aspects of usability in the home. Health-related quality of life, both the index score and the VAS score, were lower among the population younger than 75 years, those with the higher dependence in ADL, and among those with lower usability scores. Those living alone had a significantly higher index score, indicating a better health profile, but on average a similar score for self-rated health on the VAS.

The regression models are presented in Table 2. The final analysis included 174 and 178 participants, for the VAS score and the index score variables, respectively. Results show that the variance in health-related quality of life (both scores) was significantly associated with age. More specifically, participants aged under 75 years rated their health lower compared to the group of those aged between 75 and 90 years (*p* values equal to 0.016 and 0.009 for the VAS score and the index score respectively). ADL dependence was also significantly associated with health-related quality of life (both scores); the more dependent participants were in ADL, the worse their ratings on the EQ-5D (*p* values equal to 0.011 and 0.001 for the VAS score and the index score respectively).

Finally, participants who were more satisfied with the social aspect of usability in their homes also rated their health better than those who were less satisfied, using the VAS score (*p* = 0.008) Conversely, the social aspect of usability in the home was not significantly associated with health-related quality of life when using the EQ-5D index score as dependent outcome measure. Similarly, the self-care aspect of usability was significantly associated with the EQ-5D index score (*p* = 0.001) but not the VAS score.

## 4. Discussion

The aim of this study was to investigate factors associated with health-related quality of life among people applying for HA in Sweden. For this purpose, the study used multiple regression analysis to test the influence of variables already known from previous research to have an impact on health-related quality of life, including personal factors, activities, participation, and environmental factors.

The findings conclude that three variables were significantly associated with health-related quality of life measured with the VAS in this sample: age, ADL dependence, and the social aspect of usability in the home. Variables significantly associated with the health summary index were age, ADL dependence and the self-care aspect of usability.

Age in our data had a skewed distribution, and thus, was reclassified into three categories. The youngest age category covered a wide range, 25–75 years, and included 27.95% of the participants. The regression analysis showed that this age group rated their health significantly lower than the other older groups; a fact, which was not discovered when age was used as a continuous variable. Thus, categorization helped clarify the relationship between age and self-rated health in this case. In the general population, younger people rate their health higher than older people [5], but in this study, those aged under 75 years rate their health lowest compared to other groups.

A potential explanation might be that their expectations on life in general are similar to others in the same age, but when comparing their own life with others they feel much more restricted and impacted by their activity restrictions, in this study demonstrated by their higher level of ADL dependence, and thus perceive their health as lower. When it comes to the older age group, they might perceive their activity limitations and dependence as a natural consequence of ageing, and thus it has less impact on their self-rated health compared to those that are younger [20].

In our study, higher dependency in ADL was associated with low health-related quality of life. Moreover, the self-care aspect of usability was associated with a lower index score. This is in line with earlier studies focusing on older people [31,32]. A combination of functional limitations and physical demands may have negative influence on the ability to perform different activities, and therefore increase dependency in ADL [33] seems to lead to lower self-rated health [22]. An earlier study exploring the reasons for applying for a HA [34] revealed that among other things, increasing independence in ADL and not having to rely on family, friends, and home care services to perform different activities was a major argument for an application.

The social aspect of usability consisted of three items: socializing with friends and family in the home, contacting others via telephone/computer, and watching TV/listening to radio. The findings showed that participants, who were more satisfied with this aspect of usability, also rated their health higher. Social contact and possibility to engage in social activities is related to higher self-rated health in several previous studies [35,36,37]. Some of the participants in this study are considered as frail elderly and might have limited possibility to engage in social activities outside the home, which makes the own home and its usability more important.

A limitation of the study is the low representation of people with cognitive impairment, due to a higher frequency of missing data among this population. Cognitive impairment could not be assessed for 19.2% of the sample, due to fatigue, low mood, or disability among these participants. Thus, it is likely that the prevalence of cognitive impairments among persons applying for HA in Sweden might be higher than what actually is observed in our sample. In addition, only people able to communicate in Swedish were included in this study. This could be due to disability, such as aphasia or dementia related language difficulty, but also due to cultural reasons. Earlier research has showed that older immigrants might rate their health worse than citizens born and raised in Sweden [38]. This limitation as well might partially affect the generalizability of the results to the general Swedish population but does not affect the internal validity of our results and the consistency of the associations found.

It is also important to note that ADL dependence, as measured by the ADL-staircase, was calculated into sum-scores. This differs from the original version of the instrument [36] but has been previously applied in other studies [20,39]. Most important though, ratings of difficulty in activity performance are included in this study, potentially generating detailed information on the individuals’ dependence in ADL. Still, further validation of the scale is needed [40].

This study had a cross-sectional design and only investigated factors associated with health-related quality of life among people applying for HA. However, given the aim of the HA, it is possible that both health-related quality of life and the strength of these associations would change after the HA. Future research could involve a longitudinal design exploring how implementations of HA and/or a rejection of applications affect health-related quality of life and associated factors.

## 5. Conclusions

To conclude, a variety of factors seem to be associated with how people applying for HAs in Sweden perceive their health-related quality of life. Our study suggests that applicants with higher dependence in ADL and other groups, such as applicants with less than 75 years and/or those living in homes which are not well suited for supporting the activity related to self-care (in other terms, people reporting lower usability scores in the self-care component), could be at risk of poorer self-rated health. Our finding suggests that care professionals, such as the occupational therapist working in the municipalities in charge of providing the HA grants, should pay special attention towards the situation of people with these characteristics, in order to reduce the observed gap in health-related quality of life both through personalization of care and the provision of integrative services. Such approach would be beneficial also in terms of prevention of a further health decline, as the literature strongly support that differences in health-related quality of life are strongly associated with further increase of disability and disease-related care burden.

## Figures and Tables

**Table 1:** Bivariate associations between selected variables and EQ-5D measures.

Variable	EQ VAS				EQ-5D-5L Index			
	*n* (%)	Mean	SD ±	*p* *	*n* (%)	Mean	SD ±	*p* *
Gender								
Male	82 (36.61)	55.32	22.9	0.621	87 (37.02)	0.51	0.26	0.1732
Female	142 (63.39)	56.75	19.69		148 (62.98)	0.56	0.25	
Age								
<75 years	85 (37.95)	49.79	22.93	**0.001**	87 (37.02)	0.45	0.28	**0.001**
75–90 years	117 (52.23)	60.02	19.24		123 (52.34)	0.59	0.22	
>90 years	22 (9.82)	60.95	14.58		25 (10.64)	0.61	0.23	
Living conditions								
Alone	124 (55.61)	56.99	19.49	0.593	132 (56.41)	0.57	0.25	**0.048**
Together	99 (44.39)	55.48	22.58		102 (43.59)	0.5	0.26	
Cognitive impairment ^1^								
Missing value	43 (19.20)	57.88	20.98	0.644	50 (21.28)	0.56	0.25	0.512
10–17	28 (12.50)	52.32	20.88		27 (11.49)	0.49	0.34	
18–25	97 (43.30)	57.3	20.7		102 (43.40)	0.54	0.23	
26–30	56 (25.00)	55.05	21.39		56 (23.83)	0.57	0.25	
ADL dependence ^2^								
0–8 (Q_1_)	68 (30.36)	63.18	19.11	**0.006**	69 (29.36)	0.66	0.16	**<0.001**
9–11 (Q_2_)	42 (18.75)	56.57	19.86		45 (19.15)	0.58	0.22	
12–15 (Q_3_)	64 (28.57)	52.38	19.76		66 (28.09)	0.51	0.24	
16–27 (Q_4_)	50 (22.32)	51.42	23.26		55 (23.40)	0.41	0.31	
Usability-self-care aspect ^3^								
0–14 (Q_1_)	58 (25.89)	47.83	23.28	**<0.001**	62 (26.38)	0.34	0.28	**<0.001**
15–19(Q_2_)	62 (27.68)	53.39	17.73		66 (28.09)	0.56	0.23	
20–22 (Q_3_)	52 (23.21)	59.37	17.62		54 (22.98)	0.64	0.16	
23–25 (Q_4_)	52 (23.21)	65.85	20.51		53 (22.55)	0.67	0.16	
Usability-social aspect ^3^								
0–10 (Q_1_)	58 (25.89)	50.21	24.31	**0.002**	62 (26.81)	0.47	0.28	**0.014**
11–12 (Q_2_)	54 (24.10)	53.04	16.79		55 (26.38)	0.53	0.26	
13–14 (Q_3_)	61 (27.23)	57.8	21.25		63 (25.11)	0.56	0.23	
15 (Q_4_)	51 (22.77)	64.57	17.44		55 (21.70)	0.62	0.22	
Usability-outdoor/leisure aspect ^3^								
0–5 (Q_1_)	59 (26.34)	49.97	21.58	**0.002**	63 (26.81)	0.46	0.27	**0.001**
6–8 (Q_2_)	58 (25.89)	54.19	22.96		62 (26.38)	0.51	0.28	
9–11 (Q_3_)	57 (25.45)	57.18	17.18		59 (25.11)	0.57	0.22	
12–15 (Q_4_)	50 (22.32)	64.9	18.79		51 (21.70)	0.64	0.18	
Total sample	224	56.23	20.89		235	0.54	0.25	

^1^ As measured and categorized by Montreal Cognitive Assessment: moderate = 10–17, mild = 18–25, normal > 26. ^2^ As measured by ADL-staircase. ^3^ As measured by Usability In My Home. * Figures in bold are significant.

**Table 2:** Models for estimation of factors associated with EQ-5D measures.

Variable	EQ VAS (*N* = 174; *R*^2^ = 0.221)				EQ-5D-5L index (*N* = 178; *R*^2^ = 0.3792)			
	β	Std error	*p* *	(95% CI)	β	Std error	*p* *	[95% CI]
Age < 75 (with reference to age 75–90)	−7.68	3.15	**0.016**	(−13.90; −1.45)	−0.093	0.035	**0.009**	[−0.163; −0.023]
Age > 90 (with reference to age 75–90)	3.18	5.55	0.568	(−7.78; 14.14)	0.021	0.044	0.633	[−0.066; 0.109]
Sex (female)	−0.66	3.17	0.835	(−6.91; 5.59)	0.013	0.037	0.731	[−0.060; 0.085]
Living conditions (together)	0.28	2.96	0.924	(−5.56; 6.12)	−0.017	0.033	0.598	[−0.083; 0.048]
ADL dependence	−0.79	0.31	**0.011**	(−1.40; −0.19)	−0.013	0.003	**0.001**	[−0.019; −0.008]
Cognitive impairment	−0.15	0.32	0.636	(−0.80; 0.49)	0.000	0.004	0.947	[−0.009; 0.008]
Usability (self−care aspect)	0.37	0.38	0.33	(−0.38; 1.12)	0.014	0.004	**0.001**	[0.006; 0.023]
Usability (social aspect)	1.64	0.61	**0.008**	(0.44; 2.83)	0.003	0.008	0.676	[−0.012; 0.018]
Usability (Leisure/outdoor aspect)	0.31	0.42	0.47	(−0.53; 1.14)	0.004	0.004	0.393	[−0.005; 0.012]
Constant	42.35	12.97	**0.001**	(16.74; 67.96)	0.403	0.132	**0.003**	[0.142; 0.663]

β = Beta Coefficient; * Figures in bold are significant.
